# Review of machine learning methods in soft robotics

**DOI:** 10.1371/journal.pone.0246102

**Published:** 2021-02-18

**Authors:** Daekyum Kim, Sang-Hun Kim, Taekyoung Kim, Brian Byunghyun Kang, Minhyuk Lee, Wookeun Park, Subyeong Ku, DongWook Kim, Junghan Kwon, Hochang Lee, Joonbum Bae, Yong-Lae Park, Kyu-Jin Cho, Sungho Jo

**Affiliations:** 1 Soft Robotics Research Center, Seoul National University, Seoul, Korea; 2 Neuro-Machine Augmented Intelligence Laboratory, School of Computing, KAIST, Daejeon, Korea; 3 Biorobotics Laboratory, Department of Mechanical Engineering, Seoul National University, Seoul, Korea; 4 Institute of Advanced Machines and Design, Seoul National University, Seoul, Korea; 5 Soft Robotics & Bionics Lab, Department of Mechanical Engineering, Seoul National University, Seoul, Korea; 6 Bio-Robotics and Control Laboratory, Department of Mechanical Engineering, UNIST, Ulsan, Korea; 7 KAIST Institute for Artificial Intelligence, KAIST, Daejeon, Korea; CHINA

## Abstract

Soft robots have been extensively researched due to their flexible, deformable, and adaptive characteristics. However, compared to rigid robots, soft robots have issues in modeling, calibration, and control in that the innate characteristics of the soft materials can cause complex behaviors due to non-linearity and hysteresis. To overcome these limitations, recent studies have applied various approaches based on machine learning. This paper presents existing machine learning techniques in the soft robotic fields and categorizes the implementation of machine learning approaches in different soft robotic applications, which include soft sensors, soft actuators, and applications such as soft wearable robots. An analysis of the trends of different machine learning approaches with respect to different types of soft robot applications is presented; in addition to the current limitations in the research field, followed by a summary of the existing machine learning methods for soft robots.

## 1. Introduction

Soft robots have been extensively researched with respect to various research fields [1, 2]. These robots have advantages over robots made of rigid materials due to their flexibility, compliance, and adaptability to the surrounding environments [3]. Examples of their applications include soft grippers for handling fragile or delicate objects [4, 5] and mechanoreceptive or proprioceptive sensing for robot using soft sensors [6, 7]. Moreover, they are often worn on human bodies for human-robot interactions to enable safe and comfortable assistance and interaction due to their compliant structures [8, 9]. Several studies combined soft sensors and soft actuators to perform complex tasks like robot perception [10].

In spite of the advantages of soft robots, there exist common limitations in modeling, calibration, or control since the structural compliance and the viscoelasticity in the material results in complex and unpredictable behaviors due to non-linearity [8, 11, 12] and hysteresis [11, 13]. Non-linearity indicates that the relationship between the system input and the output cannot be represented by a simple linear relationship. Hysteresis can be defined as a time-dependent behavior typically shown as an output discrepancy during loading and unloading cycles. There are additional drawbacks, which include creep, drift, and high degrees-of-freedom (DOF) that increase hysteresis thus contributing to the complexity of the robot behaviors. These make it difficult to mathematically model soft grippers and calibrate soft sensors, limiting the applications of soft robotics.

A potential solution to the aforementioned drawbacks is implementation of machine learning techniques. It is well known that machine learning algorithms are effective in solving non-linear problems in various fields [14–16], and they have recently been used to solve problems related to soft robots. In particular, the applications include soft sensor calibrations [17, 18], positioning control of soft actuators [19, 20], and more complex tasks, such as grasping [21, 22] or motion planning of robots [23, 24]. Based on the studies, the use of machine learning-based methods has successfully addressed the current limitations of soft robots.

This paper presents and analyzes existing machine learning methods in the soft robotics. It aims to present an overview, analyze the current trend, and discuss current limitations of machine learning algorithms in soft robotics. Relevant studies on soft sensors and soft actuators are presented, followed by the implementation of integrated soft systems in various applications such as wearable devices, grippers, and manipulators. Furthermore, a discussion on the remaining limitations is presented, followed by the conclusion of the study.

### 1.1 Backgrounds

This study categorizes machine learning methods in soft robotics into two sections: Sensors in soft robotics and Actuators in soft robotics. The Sensors section introduces calibration and characterization methods using machine learning (2.1.1 Sensor calibration and characterization), and practical applications, such as obtaining tactile or human posture information (2.1.2 Sensing in practical uses). The Actuators section includes static/dynamic modeling and control of soft pneumatic actuators (2.2.1), cable-driven actuators (2.2.2), and Electroactive polymers and shape memory alloys (2.2.3). In section 2.2.4 Actuators in practical uses, such as wearable devices and manipulators, are introduced. Recent studies with respect to hardware types and tasks are categorized in Table 1, and the terminologies and abbreviations used in the paper are presented in Table 2.

**Table 1:** Research with respect to hardware types and tasks.

Sensors	Category	Soft Pressure Sensor		Soft Strain Sensor
		Soft Pressure Sensor Array		
	Sensor characterization/ calibration	[17, 18, 50–53]		[49, 54]
	Sensing in practical uses	[25, 26, 32, 53, 56–60]		[61, 66–68],
Actuators	Category	Soft Pneumatic Actuators (SPAs)	Cable-Driven Mechanism (CDM)	Others (EAP, SMA, etc)
	Statics/dynamics modeling (proprioception)	[10, 19, 20, 82, 84]	[102–105]	[114]
	Model-based control strategy	[85–93]	[109]	[24]
	Model-free control strategy	[83, 94, 96–98]	[100, 106–108]	[62, 111–113]
	Actuators in practical uses	Soft wearable devices	[101, 115–117]	
		Soft manipulators and grippers	[23, 118–126]	

**Table 2:** Terminology and corresponding abbreviations.

Terminology	Abbreviation
k-Nearest Neighbors	kNN
Support Vector Machine	SVM
Decision Tree	DT
Gaussian Process	GP
Gaussian Mixture Model	GMM
Gaussian Mixture Regression	GMR
Hidden Markov Model	HMM
Feedforward Neural Network	FF
Multi-layer Perceptron	MLP
Convolutional Neural Network	CNN
Recurrent Neural Network	RNN
Long Short-Term Memory	LSTM
Autoencoder	AE
Generative Adversarial Network	GAN

**Fig 1:**
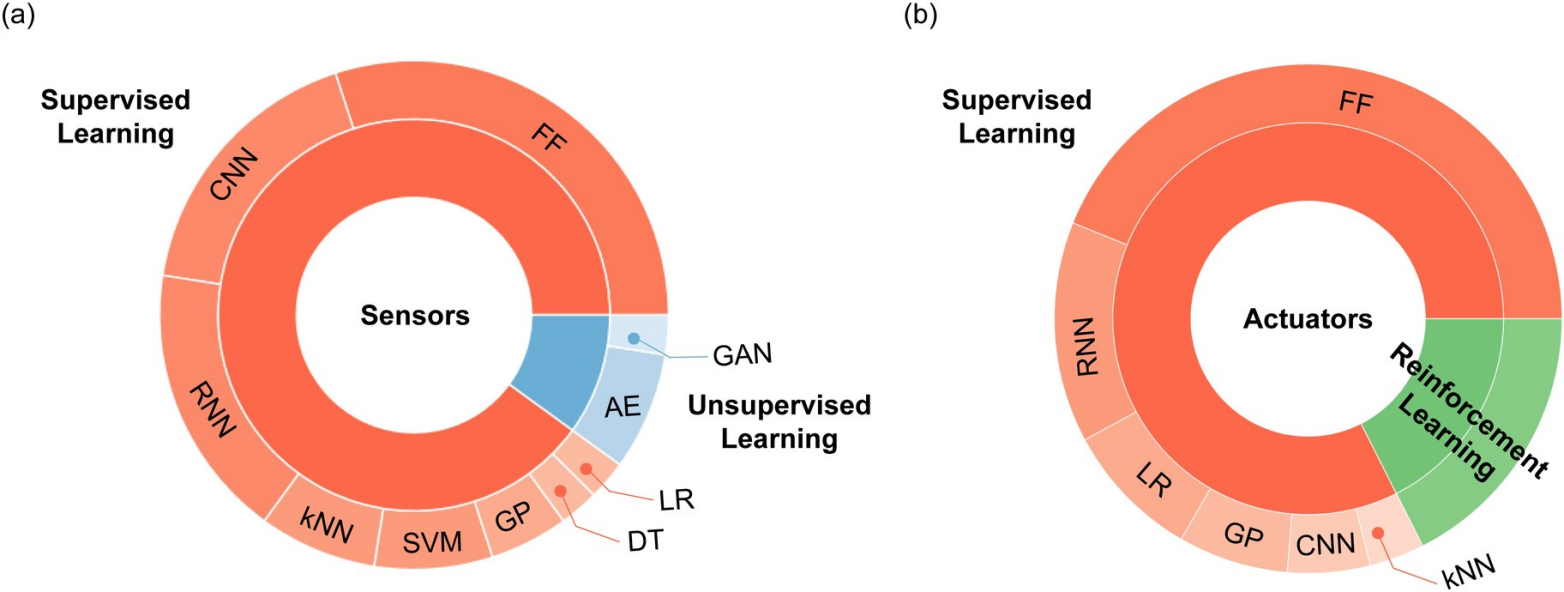
Chart for learning techniques. (A) Sensors, (B) Actuators.

Fig 1 overviews machine learning methods used throughout the papers. In sensor-related studies, supervised learning methods such as k-nearest neighbors (kNN), support vector machine (SVM), and supervised deep learning models are mainly employed. Given that these algorithms are generally employed for classification, they can be used to distinguish different objects in contact. For the calibration of current soft sensors, a recurrent neural network (RNN), a deep learning algorithm specialized in time-series data, are frequently employed. For sensors that have two-dimensional array data types, such as e-skin, a convolutional neural network (CNN), an effective deep learning algorithms for image processing, has been used, for tasks such as classification of contact objects [25] or hardness estimation by combining with an LSTM network [26].

Moreover, several reinforcement learning algorithms have been employed in studies for actuators, grippers, and manipulators. Based on the surveyed studies related to those applications, the main objectives of machine learning are to perceive the position of the devices and to control them to the desired positions. To accomplish such tasks, some papers have used reinforcement learning algorithms to control the robots. Reinforcement learning algorithms are to develop strategies or policies to learn the expected behaviors by designing reward functions. The existing studies have proposed new reward functions that are suitable for the target robots. To acquire state information of the robots, vision sensors, like a camera or Vicon, have been frequently utilized.

Overall, it should be noted that several studies utilized deep learning-based approaches. These studies reported that deep learning models can successfully address the existing issues of soft robots, such as non-linearity and hysteresis, and showed that the learning-based approaches had better performances compared to non-deep-learning-based approaches. However, a limited number of studies have been conducted based on unsupervised learning in the soft robotics research field, unlike other fields wherein unsupervised learning methods such as autoencoders and generative adversarial networks are widely implemented. The studies analyzed with respect to the employed algorithms are presented in Table 3.

**Table 3:** Research with respect to algorithms.

	Learning Type	Algorithms	Paper Lists
Sensors	Supervised Learning	kNN	[17, 49, 59]
		SVM	[49, 58, 59]
		DT	[49]
		GP	[49, 59]
		LR	[49]
		FF	[17, 49–54, 58, 61, 66–68]
		CNN	[25, 26, 32, 56, 59, 60, 68]
		RNN	[17, 18, 26, 58, 66–68]
	Unsupervised Learning	AE	[55, 60, 67]
		GAN	[68]
Actuators	Supervised Learning	kNN	[21, 117]
		GP	[24, 84, 89, 90]
		LR	[19, 20, 83, 91, 117]
		FF	[19, 20, 23, 62, 82, 85–88, 93, 94, 98, 101–105, 109, 112, 115–118, 125, 126]
		CNN	[22, 92, 116]
		RNN	[10, 101, 106, 108, 114, 116, 124, 126]
	Reinforcement Learning		[82, 94, 97, 98, 100, 107, 109, 112, 113, 117]

## 2. Materials and methods

### 2.1 Sensors in soft robotics

Soft sensors have been intensively studied as one of the crucial technologies in soft robotics to enhance the perceptivity and adaptability of robots to their surroundings by estimating mechanical stimuli and deformations similar to mechanoreceptors or proprioceptors in biology. Various soft sensors have been developed by embedding different types of electrically conductive fillers such as liquid conductors (liquid metal [27, 28], ionic liquid [29, 30]), nanomaterials (nanotube [31, 32], nanowires [33, 34], nanocomposites [35, 36]), and conductive fabrics [37, 38] into soft structures composed of elastomers. Consequently, soft sensors can detect large deformations such as strain, curvature, and compression by measuring electrical changes of the fillers such as the resistance [39, 40] and capacitance [41, 42].

A major limitation when using soft sensors is the complexity of their characterization and calibration. This is caused by the hyper-elastic characteristics of soft materials cause non-linearity, large hysteresis, creep, and drift, resulting in generating unexpected physical behavior and electrical responses of soft sensors. These drawbacks make use of soft sensors more difficult than that of traditional sensors. Therefore, several studies have been conducted to find solutions through the modifications of hardware design [43, 44] or based on empirical approaches to calibrate soft sensors [18].

Moreover, soft sensors have been integrated with other robotics technologies, such as actuators, grippers, manipulators, and wearable devices, to better understand their physical interactions with environments or their own physical states. Soft sensors attached to or embedded in grippers estimate the magnitudes and locations of contacts. Furthermore, they are not only able to estimate types of materials and shapes of the gripped objects but also able to detect the slippage of objects based on the analysis of contact information extracted from post-processed sensor data. In the cases where array-type soft pressure sensors are used in soft mobile robots and soft manipulators, they can estimate the positions and motions of the robotic systems as well as the distribution of interaction forces during contact. When soft sensors are used for soft wearable devices by attaching to the human body, the sensors estimate body motions such as upper limb motions, gait, or hand motions without physical resistance due to their softness and elasticity. These tasks, however, can introduce additional non-linear behaviors to soft sensors due to the non-linearity of human body.

**Fig 2:**
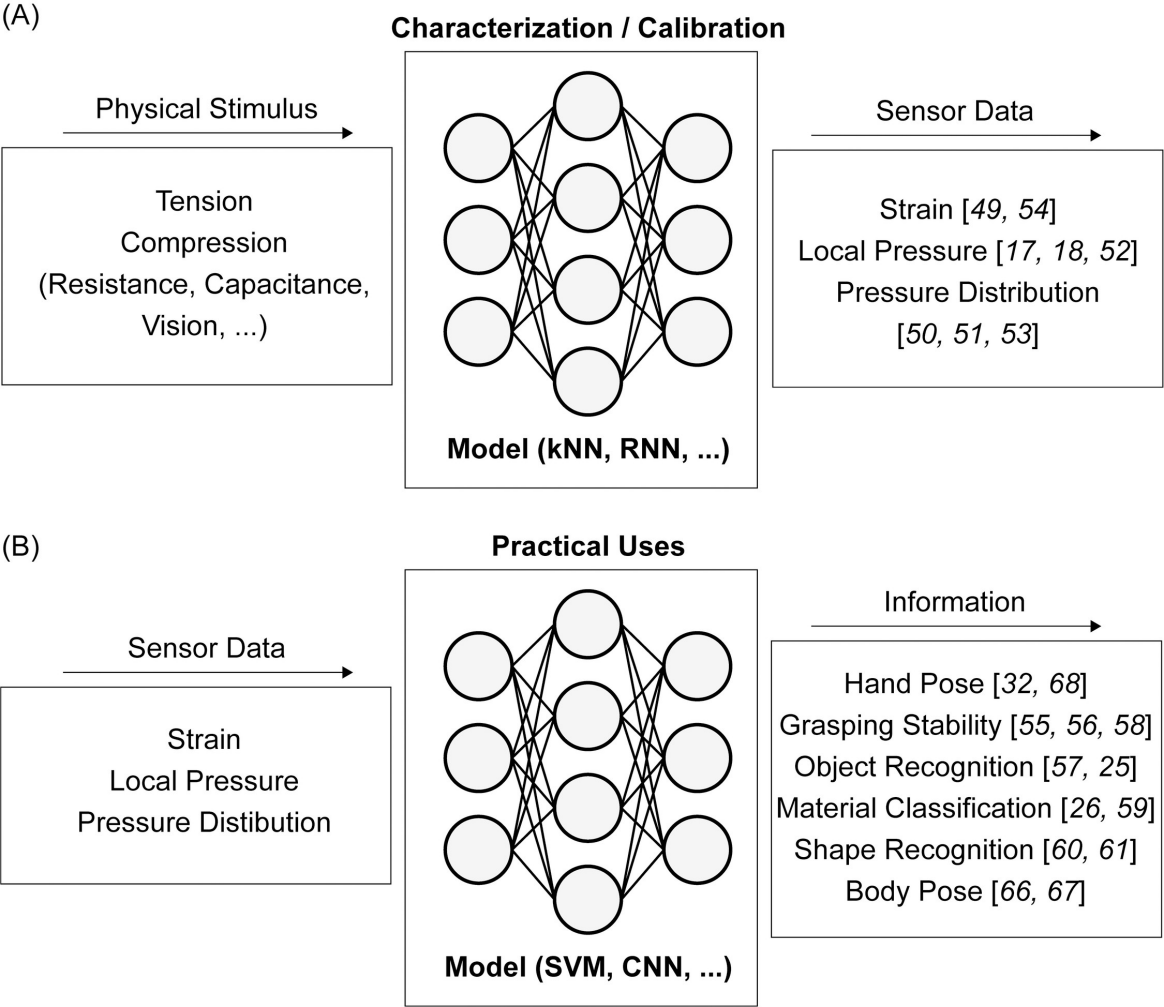
Model Inputs and outputs of soft sensors with machine learning methods. (A) Sensor calibrations and characterizations, (B) Sensing in practical uses.

To overcome such limitations and implement better uses of soft sensors, nowadays, learning-based approaches are actively used as one of the most effective empirical methods. Machine learning can accurately characterize and calibrate soft sensors by taking into account their nonlinearity and hysteresis, which are not easily represented using analytical and experimental approaches. Moreover, when multiple or array type soft sensors are used for some purposeful tasks such as estimating body motions and grasping characteristics of interacted objects, learning based approaches efficiently process massive and non-intuitive datasets from sensors, to extract meaningful features and information required for completing the tasks. Fig 2 depicts papers that are related to soft sensors and learning-based approaches.

#### 2.1.1 Sensor calibrations and characterizations

The main objective of sensor calibration is to accurately estimate the physical stimuli such as pressure or strain applied to soft sensors. However, in the calibration process, a large hysteresis loop of output signals during loading and unloading cycles increases the complexity of traditional analysis and experimental methods. Therefore, the hysteresis characteristics have been extensively researched using mathematical models and data-based approaches [45]. The parameters of hysteresis model in the data-based approaches are generally optimized using a machine learning algorithm as well as convex optimization methods.

Nowadays, deep learning is widely implemented due to its effectiveness in massive data processing in research fields, such as computer vision [46] and natural language processing [47]. With respect to soft sensors, some studies have been also conducted based on deep learning methods, which have been compared with non-deep-learning methods to evaluate the performances of the proposed model. Navarro et al. used FF and a transfer learning approach to obtain a contact location and to estimate the corresponding pressure applied to a soft pad and a kidney-shaped sensor, which are soft pneumatic mechanosensors made of silicone. With the proposed learning methods, the change of volumes using airflow sensors were measured and calibrated to estimate magnitudes of applied forces. They compared the estimation results obtained using the learning-based approaches with those from numerical methods like the online finite element method. According to the comparison, the neural network model provided better performances in obtaining contact forces, but the algorithm was not accurate in unobserved force ranges [48]. Given that sensor data are sequential in time; RNNs, specialized for time-series data, are considered as a suitable network for the calibration of soft sensors. Kim et al. proposed methods to estimate the magnitudes and locations of contact forces applied to soft pressure array sensors based on the Preisach method and ANNs [11]. In the case of single contact, localization was conducted using a kNN, and a general RNN was used to extract the temporal characteristics for the analysis of the hysteresis. The performance was then compared with that of an ANN model that determines the parameters of the Preisach method. In addition, this study showed that multi-contact localization was possible using only a simple logic. It also showed that both magnitude estimation and localization of multi-contact can be conducted with long short-term memory (LSTM), which is a type of RNN that is applicable to long-range dependencies [46], and fully connected layer. In a similar manner, to predict the magnitude and location of pressure applied to a soft microfluidic pressure sensor, Han et al. also proposed an RNN model based on LSTM [18]. The non-linearity of soft sensors, which includes significant hysteresis, was successfully modeled using a RNN that extracts temporal features. The outputs from the RNN model were then used as inputs of the fully connected layer to predict both the magnitude and location of the pressure applied to the sensor.

In addition, there are various soft sensors which embed multiple sensing elements in a single sensing structure to detect multiple deformation modes, force distribution, or forces in multi-axes. In these cases, for easier and more efficient calibration processes, machine learning algorithms have been used as powerful tools. Van Meerbeek et al. employed several learning methods to calibrate a soft sensing structure embedding with multiple optical fibers [49]. Physical behaviors such as bending and twisting of the silicone matrix were estimated using kNNs, SVMs, and decision trees for the classification of deformation types. In addition, kNNs, SVMs, decision trees, Gaussian processes, linear models, and ANNs were employed as regression models for the estimation of bending and twisting angles, and the accuracy of each model was compared. kNN showed the best performance with low average error and model bias. Sohn et al. developed a macroscale soft pressure array sensor using a multi-walled carbon nanotube/polydimethylsiloxane (MWCNT-PDMS) composite film, which was of a single-layered piezoresistive type [50]. Using a deep learning technique, pressure distribution can be estimated based on the single-layered simple composite film. For contact localization, a network with 36-dimensional output nodes in three fully connected layers was used, and a network with one-dimensional output nodes in two fully-connected layers was employed for the magnitude estimation of the applied pressure. Park et al. also conducted a real-time estimation of contact force distribution detected via a soft tactile sensor using an electrical impedance tomography (EIT) sensing method through a DNN [51]. It should be noted that the traditional signal processing methods using the linearized models, such as Maxwell’s equation, was limited with respect to construction accuracy while it showed fast computation time and robustness against noise. In contrast, the proposed nonlinear EIT algorithm implemented based on the deep neural network was able to improve both reconstruction accuracy and computation time of EIT sensing. Chuah et al. developed a soft three-axis force sensing footpad by embedding nine commercial air pressure sensors in a silicone composite [52]. Given that the complex geometry of the developed sensors and the use of elastomers limited the analytical modeling of the sensor, the sensor was successfully calibrated with an ANN based on ground truth three-axis force data collected by the contact between the footpad and three-axis force sensor in various directions.

To use multiple numbers of sensors or array sensor, each of the individual sensors or sensing units need to be calibrated independently; this consumes lots of memory and network weights. To mitigate this difficulty, when Sferrazza et al. conducted a study on the reconstruction of the normal pressure distribution using a vision-based tactile sensor and an FNN [53], transfer learning was used to transmit the data among multiple sensors. This learning method was able to reduce training time and efficiently process large dataset, while maintaining superior sensing performance. Kim et al. also used an optimal transportation transfer learning to learn the model of soft sensors with large volume [54].

#### 2.1.2 Sensing in practical uses

In the previous section, we introduced studies that efficiently and accurately calibrate soft sensors using machine learning techniques. This section deals with applications aimed to perform purposeful tasks based on tactile or human-related information obtained from sensor data other than just calibrating sensors.

First, soft sensors have been widely employed to obtain tactile information, such as single- or multi-point contact pressure, vibration, during physical interactions with the environment by mimicking the functionalities and properties of skin. The tasks that involve the use of soft tactile sensors are not limited to contact localization and magnitude estimations. They also include extended applications, such as contact stability estimation, object type or shape recognition, and material classification, especially when they are integrated with grippers. Since these tasks need to process large and non-intuitive sensing datasets to extract meaningful features and required results, various appropriate machine learning techniques have been actively applied. To recognize objects in contact, classification algorithms such as SVM and kNN are used. Given that typical soft tactile sensors are composed of multiple sensing nodes like human skin, the data collected from the sensors are similar to multi-dimension image data. Hence, a CNN, which is one of the deep learning algorithms that are specialized with respect to image processing, is generally used.

Roberge et al. conducted a study on the classification of gripping states using a soft pressure sensing pad, to establish whether contacted objects were stably gripped or subject to slippage based on magnitude and frequency information of contact force. Sparse coding, which is a statistical model that can be learned using only a small amount of data, was used to train the classifier with the sensing data. The classifier was then re-trained using SVM based on the initial training results. Thereafter, the gripping states were estimated [55]. Larson et al. proposed a soft tactile interface that can recognize human gestures and contact location based on a capacitive-type tactile sensor array made of stretchable carbon nanotube dielectric elastomer embedded in a rubber membrane [30]. To determine the features including gestures and contact locations, the sensor data were trained using a three-dimensional CNN (3D-CNN) model for gesture recognition, and a 2-dimensional CNN model for contact localization. Calandra et al. attached GelSight high-resolution pressure mapping sensors to a fingered gripper for the analysis of the tactile information upon contact between the gripper and the object [56]. Then, the efficient and stable grasping adjustment for the most promising grasping motions was predicted through the proposed end-to-end action-conditional model based on a deep multi-modal convolutional network. The model overcomes the limitations of traditional gripping strategies that are primarily dependent on visual information. It provides a strategy for reliable gripping without the requirement of complex sensor calibrations or analytical contact force modeling. Zimmer et al. also conducted a study to estimate effective grasping of a shape-memory actuated gripper using multiple machine learning methods such as LSTM, SVM, spatiotemporal hierarchical matching pursuit (ST-HMP) [57], and a feed-forward neural network (FNN) [58].

Furthermore, Yuan et al. estimated the shore hardness of contacted objects by obtaining features of image frames based on pressure distribution data via the GelSight soft sensor by using a CNN and LSTM [26]. Baishya et al. conducted a study on material classification by attaching a flexible tactile skin to a robot hand. They used a CNN algorithm, whose performances were then compared with those of several learning algorithms upon two datasets that have different features [59]. A pressure-mapping sensor (Tekscan; Grip VersaTek 4256E) was used to gather spatiotemporal signals. Six types of materials were classified using CNN. Thereafter, the performance of proposed CNN algorithm was compared with those of various classification algorithms including Gaussian classification, kNN, and SVM; the CNN algorithm showed better classification accuracy. Polic et al. conducted a study to determine object shape, edge position, orientation, and indentation depth information required for object manipulation using an optical-based tactile sensor (TacTip) attached to the end effector of a robotic arm based on a CNN algorithm [60]. The main contribution of this study was the implementation of an unsupervised feature extraction method using a CNN autoencoder. This model allowed for the extraction of sufficient features from a small size dataset in addition to rapid model training due to its simple architecture. Masaki et al. conducted a study on the estimation of surface undulation using a strain gauge and an artificial neural network [61]. A system for the estimation of the surface undulation was then implemented by attaching the strain gauge covered with the silicone rubber layer to an index finger. The signal from the strain gauge was pre-processed and inputted into an FNN for the estimation of the surface undulation levels.

There also are various cases that multiple soft sensors are used in wearable devices to recognize human motions. Soft pressure sensors have been attached to soft gloves and insoles to recognize gripping states and walking motions by detecting in-contact with objects or ground. Soft strain sensors primarily estimate upper or lower limb motions, gait or hand motions by being attached to the joints with a single DOF or multiple DOF, i.e., finger, elbow, shoulder, knee, and ankle joints [62–65]. For these applications, the data obtained from soft sensors are correlated with the human biomedical and kinematics information such as the gait pattern. However, the relationship is not linear, thus increasing the complexity of the modeling and sensor calibration. Learning-based methods have been recently proposed to overcome such limitations.

Kim et al. proposed a deep full-body motion network (DFM-Net) for calibrating human motions. In the study, using a wearable sensing suits with 20 strain sensors; an encoder-decoder structure was proposed for encoding sensor information based on LSTM, and the decoding kinematic information using an FNN [66]. Kim et al. also proposed a gait motion generation method based on two multi-fluidic soft strain sensors [67]. The objective of the algorithm is to decrease in the amount of data based on a semi-supervised approach. In particular, the gait motion was embedded using an autoencoder, and decoded using an FNN.

Various studies were also conducted related to human hand. Glauser et al. employed neural networks for the analysis of strain sensor data and the recognition of hand motion [68]. In particular, various deep learning-based algorithms, which included a fully convolutional network (FCN), LSTM, residual neural network (ResNet) [69], U-Net [70], and conditional generative adversarial network [71] were used; and U-Net yielded the highest accuracy with respect to the reconstructions of hand motions. In addition, Sundaram et al. conducted a study related to grasping using a scalable tactile sensor glove with 548 sensors [25]. They estimated the grasped objects, and their weights were determined using a CNN. It also explained the key correspondences of different human hand regions by measuring tactile patterns during grasping.

#### 2.1.3 Sensors: Limitations and future works

Good learning results can be caused by well-trained learning models using sensor datasets with consistent signal patterns and ranges. However, since soft sensors have manufacturing tolerances for several reasons, such as variations of elastomer properties and manufacturing human errors, even homogeneous sensors have variations of characteristics, resulting in performance variations, such as different initial offset and operating ranges. In addition, test conditions such as the size of an indenter and clamping types can make a sensor behave differently; output data can be susceptive to change even if input data are the same. In this case, even if a model shows excellent learning results based on datasets from one specific sensor, the model cannot be applied to other sensors. In addition, since sensors made of soft materials are not durable enough for long time usage, drift can occur in sensor response as sensor structure is permanently deformed. Although there are some learning approaches such as transfer learning [53, 54] and multi-domain learning to address such limitations, improvements in sensor hardware aspects of sensing mechanisms, materials, and manufacturing processes must be accompanied for fundamental solutions.

Machine learning has its ability to extract important features from massive and multi-dimensional data. This enables researchers to design new types of soft sensors based on novel mechanisms while minimizing the concerns of dealing with sensor behaviors that can be difficult to analyze using analytical models. There are typical examples such as a multi-axis force sensor using a silicone matrix embedding multiple biometers [52] and tactile sensors capable of detecting contact forces and shape of contact objects by analyzing silicone surface images using camera sensors [53, 56, 60]. Since these novel sensors have a hardware design or a sensing mechanism that makes sensing datasets more complicated, they cannot be easily developed due to the limitation of data processing methods until activation of the use of machine learning techniques. Therefore, by understanding the characteristics of various learning approaches and taking advantage of an appropriate machine learning technique, researchers can try more various sensor designs and mechanisms to develop novel sensing structures without concerns about data processing. This shows one of the technical synergies that the learning-based approaches and soft sensors can create in the future.

### 2.2 Actuators in soft robotics

**Fig 3:**
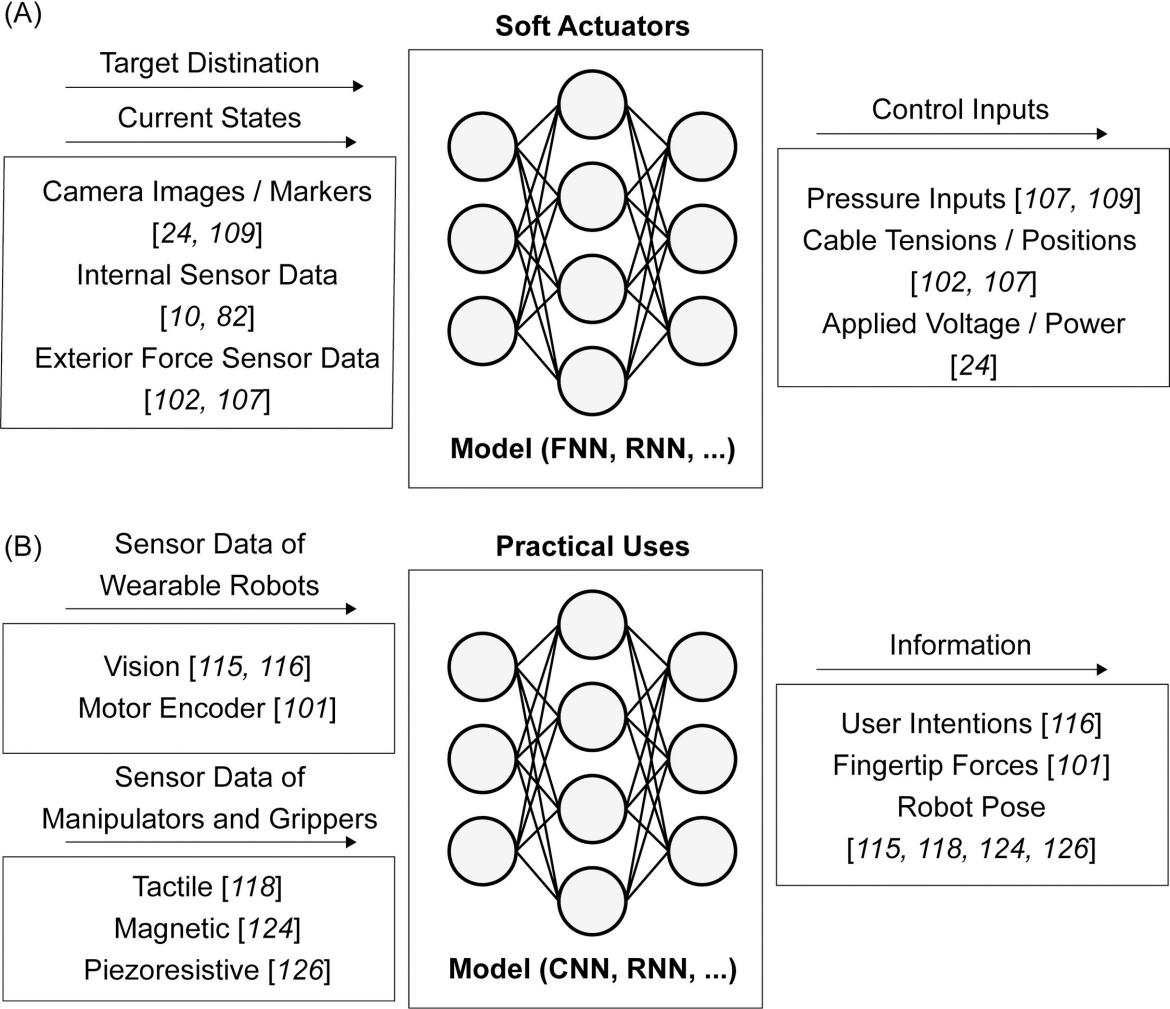
Model inputs and outputs of soft actuators with machine learning methods. (A) Actuators, (B) Actuators in practical uses.

Soft actuators are often combined with rigid robot bodies or embedded in soft robots to control them. Soft actuators are mainly categorized as pneumatic actuators (SPAs) [72–74], cable-driven actuators [75], electroactive polymers (EAPs) [76], and shape memory alloys (SMAs) [77–79] based on their actuation methods. Fig 3 depicts the inputs and outputs of machine learning models used in soft actuators.

Due to the high degree of freedom of hyper-elastic materials [80], it is difficult to realize accurate proprioception or control of soft robots using soft actuators. To control them, high-dimensions of soft morphology should be actuated with less control inputs. In addition, time-varying material characteristics limit the dynamic modeling of soft actuators. In detail, the degradations of soft matter, i.e., creep, fatigue, and friction, known as critical factors of time-varying material characteristics, are often occurred, which limits the dynamic modeling of the soft actuators. For example, frictions between the cable and cable sheath in a cable-driven approach make cable tensions highly fluctuate, which in result increases the hysteresis of cable-driven actuators and shortens the lifetime [81]. At present, machine learning methods have been extensively applied to the modeling of soft actuators that have high degree of freedom and to the generating control strategies in order to deal with the aforementioned non-linearity issues.

This section introduces existing machine learning-based researches conducted on soft pneumatic actuators and cable-driven actuators, among other actuators such as EAPs and SMAs.

#### 2.2.1 Soft pneumatic actuators

Soft pneumatic actuators (SPAs) have been extensively researched due to their flexible motions with simple morphological structures and versatility. To improve the functionality of SPAs, various sensors have been integrated for training data obtained from soft pneumatic actuators. Given that the solid-state sensors traditionally used in rigid robots may limit the flexible movements of SPAs, soft and flexible sensors have frequently been integrated to obtain contacts or bending motions of SPAs. In addition, the simple internal pressure sensor data of the SPA was used to improve the functionality of the soft gripper [82]. RNNs were employed for the SPAs, which were integrated with soft resistive sensors to obtain the contact forces and the bending motions [10]. Instead of embedding the sensors into the SPAs, a camera sensor was used to obtain the states of the actuators. To track the 3D trajectories of the SPA, an inverse model was also employed for training, as the application of the nonparametric and online learning of locally-weighted projection regression for endoscopy applications [83]. Jung et al. developed a proprioceptive sensing method of a soft pneumatic actuator based on the GP regression by incorporating with an extended Kalman filtering for state estimation and sliding mode control for the feedback control strategy [84].

Obtaining a kinematic or dynamic model of a soft robot has been a challenge in model-based control strategies. To overcome such limitation, learning algorithms have been applied to acquire the kinematic or dynamic model of soft robots based on SPAs [85–88]. An FNN and radial basis function (RBF) neural networks were applied to the inverse or forward kinematic modeling of a soft continuum robot based on SPAs including 3-Dimensional motions [85, 86]. M. Gillespie et al. and P. Hyatt et al. proposed a predictive model based on the neural networks, and a learning method for the linearized discrete state space representation of soft robots [87, 88]. G. Fang et al. developed a learning method based on the local Gaussian Process Regression (GPR) to estimate the motion of SPAs using the kinematic model from the control inputs to the manipulator configurations based on the sequential camera images [89]. Instead of the inverse, or forward kinematic modeling, an asymmetric hysteresis of a pneumatic artificial muscle was modeled by integrating the Convolutional Neural Network and an existing extended up-parallel Prandtl-Ishlinskii model. J. Kim used a Gaussian Process Regression to learn control policy for a simple tripod mobile robot based on membrane vibration actuators [90].

M. Rolf et al. developed learning strategies to obtain an inverse model, which indicates the relationship between the target position and the required control inputs [91]. Instead of modeling the dynamics of a soft robot itself, hysteresis was also predicted for a pneumatic artificial muscle over a wide range of input by combining conventional hysteresis model and the CNN [92]. M. Wiese et al. studied hyperparameter optimizations to model SPAs using a simple FNN [93].

Another approach for controlling the pneumatically actuated soft robot is a model-free learning algorithm, which is a learning method to calculate the control policy without an analytical model. Reinforced learning algorithms such as Q-learning have been usually used for the model-free approaches [94]. In general, the objective of reinforcement learning is to find the control policy that maximizes the expected discount return, which is the weighted sum of rewards received by the agent for the system [95]. X. You et al. and S. Satheeshbabu et al. developed and implemented a multi-segment soft manipulator for planar motions using the Q-learning algorithm [96, 97]. J. Kim et al. used a model-free reinforcement learning algorithm to control a pneumatic actuated tripod mobile robot. They used an adaptive soft actor-critic (ASAC) algorithm and a reinforcement algorithm to obtain an accurate dynamic model of the robot [98].

Commercially available sensors, like depth cameras, film-based flex sensors, and potentiometers, are generally used to estimate the configurations of SPAs with machine learning techniques [85, 88, 89, 91]. On the other hand, as the traditional sensors can be relatively too rigid to be compatible with SPAs that are highly deformable, soft sensors have often been integrated with SPAs to estimate the configurations of soft robots. However, the non-linear behaviors of the soft sensors may cause delays when estimating states of soft robots. For example, T. Thuruthel et al. suggested SPAs integrated with a soft sensor using cPDMS (carbon-polydimethylsiloxane) and film-based flex sensors to estimate contact forces or configurations; they reported that the proposed learning-based model showed longer delays when using the soft sensors compared to the film-based flex sensors due to the high-dimensional deformability of soft sensors [10]. Based on this perspective, it would be an open issue for the future direction to develop soft sensors with fast responses. For instance, the development of three-dimensional printing-based fabrication of soft sensors would be a possible solution to estimate the configurations of SPAs in that it tends to have consistent and fast responses [99]. At the same time, machine learning algorithms need to be developed to overcome the nonlinear dynamic responses of soft sensors when integrated with SPAs. Although [92] showed potentials in that the large hysteresis of the SPAs could be reduced via machine learning algorithms, it was limited to simple linear motions of Pneumatic Artificial Muscles (PAMs). Thus, it is necessary to develop algorithms that deal with the non-linear and hysteresis behaviors of soft sensor-embedded systems with fast responses, as a future goal.

#### 2.2.2 Cable-driven (tendon-driven) actuators

In cable-driven or tendon-driven soft robots, the actuators are situated outside of the robot structures; therefore, they do not interfere with movements of the soft bodies. Instead, cables connected to the actuators transmit the tensions through the cable paths or routings, which are embedded in a soft structure [100]. When it comes to controlling the soft robots, a major problem for the cable-driven mechanisms comes from non-linearity and hysteresis. These issues are mainly caused by high friction between a cable and cable path due to tension of the cable and the bending of the cable path [101].

A supervised learning-based method was proposed with respect to soft manipulators as a solution of the inverse statics problem to realize effective grasping. M. Giorelli et al. implemented an FNN for non-constant curvature manipulators to solve inverse kinematics [102, 103]. The performance of the FNN-based model was experimentally tested by comparing with model-based numerical approach and Jacobian-based method, which requires numerical resolution of integrals along the structure as proposed in [104, 105], for a conical soft manipulator driven by two cables. Based on the results, the FNN showed better performances and faster convergence than the model-based numerical method; however, FNN required model optimization and a bigger dataset [102].

Model-free control strategies based on RNNs were developed to learn the dynamics of robots. For a soft robot with friction-manipulation mechanisms driven by a motor-tendon combination that is capable of terrestrial locomotion, the model-free control framework was experimentally applied to the robot designs while changing the shape of tendon paths, friction mechanisms, and environmental conditions [100]. Nakajima et al. demonstrated a soft silicone arm system that can be employed to deal with the transient dynamics of the soft materials based on RNN and suggested its applicability to a real-world problem [106]. Ansari et al. conducted a study on a soft robot arm module actuated by tendon-based and pneumatic-based actuators for a bathing task for elderly people. Model-free control using reinforcement learning was developed to simultaneously increase the stiffness and positioning capacities [107]. Thuruthel et al. tested a tendon-driven soft manipulator under a simulated environment, in addition to a pneumatically-driven soft manipulator, using model-based reinforcement learning for closed-loop dynamic control. For the forward dynamic model, an RNN was used. Based on the learned dynamic models, a trajectory optimization was implemented to develop an open loop controller; however, the authors reported that the open loop controller is not robust against external disturbances [108]. To overcome this limitation, a model-based policy learning method for the closed-loop dynamic control of a soft robotic manipulator using an RNN was proposed. The representation of the policy architecture allows for the stability of the controller with respect to changes in the control frequency, sensory noise, and dynamics. With respect to the simulation of tendon-driven soft manipulators and experimental evaluations of under-actuated pneumatically-driven soft manipulators, sufficient accuracy levels were maintained, and the control frequency was decreased by a maximum factor of 5 [109].

Previously, machine learning in cable-driven or tendon-driven actuators of soft robots was focused on increasing the performances of position control. Rather than position control, soft manipulators and soft wearable robots with cable-driven actuators require end-effectors’ force or cable tension to generate proper contacting forces in accordance with various object characteristics. However, due to the non-linear characteristics of friction and fatigue with the cable and the cable path, degradations of the cable over time under various loads and situations is still limited in soft robotic systems. As future works, a real-time applicable learning method should be developed by collecting time sequence data of cable tension and the configuration of the soft robot to estimate the precise force control of the soft robots [101].

#### 2.2.3 Electroactive polymers and shape memory alloys

Ionic polymer-metal composite (IPMC) flexible actuators are generally composed of ion exchange polymer films, with electrodes on both sides, which have relatively low voltages (< 4 V), can generate large strains (> 40%) and are capable of sensing and actuating under harsh conditions [110]. However, the IPMC materials have time-varying performance changes and mechanical hysteresis as well as high maneuverability and agile capabilities, thus making it difficult to plan paths of IPMC manipulators. H. Wang et al. implemented a six-segment IPMC flexible manipulator; the paths were encoded using a Gaussian mixture model (GMM). Moreover, the recommended paths were generated using Gaussian mixture regression (GMR). The verification of the learned paths was conducted using an IPMC manipulator. They reported that the data from the operator were required, the generalized trajectories from the GMM and GMR could not always ensure the complete reproduction of the demonstrated task, and the approach was effective under static environments [24]. J. D. Carrico et al. presented machine learning with Bayesian optimization for the effective motion control of 3D-printed soft IPMC actuators in a soft crawling robot platform. However, there were challenging issues when it comes to controlling IPMC actuators. First, performance degradation occurred when the actuator operated such that the current voltage was higher than electrolysis voltage of the hydrating solvent. Second, the performance of the conventional control methods deteriorated over time. Thus, future works in controlling IPMCs will be predicting and planning the performance degradations using real-time degradation data [111].

Dielectric elastomer actuators (DEAs), consist of thin elastomer membranes between two compliant electrodes, are known to have rapid responses, large voltage-induced deformations, and noise-free operations. However, viscoelastic materials of the DEAs exhibit complex time-dependent behavior, such as creep, hysteresis, and the Maxwell stress that is related to the deformation of the actuators. As a result, the actual actuations based on the electromechanical coupling are very non-linear and time dependent [112]. In the case of a cuttlefish robot with a DEA as the jet-actuator, reinforcement learning algorithms such as Q-learning were used as the actuation strategy. The experimental results verified that the optimized control using reinforcement learning can enhance the actuation performances [113]. Li et al. conducted a study on DEA control. Based on deep reinforcement learning, a model-free method can be employed to achieve the dynamic feedback control of DEAs under the consideration of their time-dependent characteristics. Experiments were conducted on circular and rectangular DEA configurations to test their accuracy and robustness with respect to changes in the material properties and structures [112].

Shape memory actuators (SMAs) generate relatively large displacements and high force/weight ratios. However, SMAs have difficulties when modeling and controlling them when the space is continuous because the relationship between strain and temperature is hysteric and changed abruptly [113]. Recent studies that involved neural networks on SMAs were focused on SMA identification and modeling [114]. C. Cheng et al. proposed an SMA-actuated multiple-DOF soft robot with a simplified adaptive neural network control algorithm for the improvement of the accuracy of position control [62].

#### 2.2.4 Actuators in practical uses

Several applications implementing soft actuators have aimed to perform tasks other than calibrations, control, or proprioception. For instance, soft wearable devices were employed to obtain body poses or fingertip forces due to contact. In such tasks, the human-related applications increase the complexity of soft robots with additional non-linearity, which can degrade performances. In addition, the human-related applications are complex for several reasons. First, human physical factors are different from person to person, like the height, weight, muscle strength, and patterns of human motions. Second, there are several different muscles involved when generating a single motion.

In several studies, learning-based methods were proposed for the manipulation of wearable hand robots. Ha et al. realized the position control of a soft wearable glove with pneumatic actuators using pressure and vision data [115]. In particular, deep learning allowed for position control in an open-loop without prior knowledge such as the user characteristics. Kim et al. proposed VIDEO-Net for the detection of human grasping by the recognition of arm behavior and hand/object interactions using a first-person-view camera [116]. The performance of VIDEO-Net was verified using a soft wearable hand robot for disabled people. Kang et al. proposed a learning-based fingertip force estimation method for wearable hand robots based on the tendon-sheath mechanism. In addition, a bending time-gradient LSTM (BT-LSTM) was proposed to mitigate the influence of the factors that decrease the accuracy of fingertip force estimations: (1) non-linearity and hysteresis of wearable robots and human hands, and (2) dynamic angular changes in the tendon-sheath [101]. Schlagenhauf et al. tested LR to control a tendon-driven soft robot hand, Cyberglove. They compared learning-based approaches, including kNN, LR, FNN, and deep reinforcement learning, when controlling soft foam robot hands; they found that kNN outperformed the other three methods under the simulated environment [117].

For soft manipulators and grippers, machine learning algorithms are primarily employed to obtain proprioception and control the robots to desired positions. Unlike rigid robots, soft robots have a high number of DOFs; thus, they are difficult to model and control. To solve this problem, machine learning models are extensively used. In particular, reinforcement learning-based methods are primarily applied, unlike other soft robotic fields. Scimeca et al. utilized an FNN to learn tactile image information. Moreover, an integration system with a tactile sensor was proposed to obtain internal pressure distributions based on the neural network [118]. In [23], a neural network controller for continuum robots was proposed. The controller comprised an FNN controller and a nonlinear feedback controller for the manipulation of an OCTARM VI manipulator [119–121]. You et al. proposed a Q-learning method for the control of a honeycomb pneumatic network (HPN [122]) manipulator. Satheeshbabu et al. proposed an open-loop position controller based on deep reinforcement learning for a manipulator (BR^2^ manipulator [123]). Watson and Morimoto proposed to localize the tip of soft continuum robots that have potential to be usable as medical devices in which the medical field needs accurate control to guarantee safety. They used a LSTM to localize the magnet at the tip of the robot compared to existing analytic and hybrid methods [124]. In [125], a hybrid model for controlling a modular collaborative Variable-Stiffness-Link (VSL) robots has been proposed. It consisted of forward kinematics and inverse kinematics whose models are 7-layer FNN. The open-loop model was compared with a traditional model-based method, and showed that their model outperformed the traditional model. [126] proposed a learning-based approach for proprioception of three-dimensional soft sensorized robots. Unlike existing studies, it uses embedded sensor information. It also predicts 3-dimensional configuration of the robots based on the sensor data. The paper used LSTM, which was compared with 2-layer FNN, and showed that the RNN-based model reasonably estimates the steady-state configuration of the soft robots.

#### 2.2.5 Actuators: Limitations and future works

Due to the aforementioned material characteristics, it is difficult to analytically or empirically model soft actuators using traditional methods, thus making it difficult to design controllers. On the other hand, machine learning methods have been used to control soft actuators with reliable results in limited workspaces. A major disadvantage of using machine learning in control, compared to physical models, is the requirement of large number of datasets. For example, when it comes to reinforcement learning, it requires a lot of rollouts to train the algorithms to obtain desired controller policies.

Overall, soft actuators commonly show mechanical hysteresis and functional degradation over time. When soft actuators are employed in robotic applications, reliability is a dominant issue. Soft actuators are made of soft materials; these materials are highly non-linear compared to rigid materials, such as large distribution of elasticity and high dimensionality. This leads to a difficulty to predict an appropriate lifetime of the model [127]. Thus, as a future direction, applying a prognostic method will be useful to estimate the performance and the lifetime of soft actuators for the practical implementations [128, 129]. Since the data-driven approaches are widely spread in prognostics field due to its ability of quick implementations and developments, machine learning will be an applicable tool to predict the time-dependent nonlinear performance of the soft actuators. 

## 3. Discussion

Although machine learning algorithms attempt to overcome the limitations of soft sensors and actuators that cannot realize accurate calibrations and controls, there are several remaining limitations to their applicability. First, machine learning methods are data-driven approach, which generally require a large amount of data for the training of their networks. The collection of large quantities of data result in significant time consumption and considerable computational load. In addition, the collected data may be unreliable or biased (i.e., the data does not represent a robot’s whole behavior but only parts), which minimizes the reliability of the results.

To solve the problem, there can be several approaches. First, using simulations enable collecting a large quantity of data in various environments. In the robotics field, simulation environments [130, 131] have already been used to reduce trial errors that may cause problems like damaging the robots. There also are soft robotic simulated environments [132–134]. However, it still remains unclear whether or not the simulated environments are useful to reduce training data in real world environments. Because soft robots tend to have a large number of degrees of freedom due to their non-linear characteristics, simulation environments need to be verified in order to be used in real world environments. In addition, there are discrepancy between mathematical or mechanical models in simulated environments and actual behaviors of soft sensors and actuators. Several papers have proposed simulation-to-real world mapping methods [135, 136]; this approach needs to be verified under soft robot environments.

Using machine learning techniques can be another solution to reduce the number of data. For example, Meta learning [137] algorithms have been proposed to learn quickly with fewer number of new data. Transfer learning aims to improve the learning process of a data by transferring information from the data from a related domain [138]. These methods have been adopted in robotic applications, i.e., by learning policies or control strategies from one human demonstrations [82, 139] or from predicted video scenes [140] for robot arms. These strategies can be used to train robots that consist of soft sensors and actuators. Moreover, these approaches can also be applicable to sensor-to-sensor calibrations or actuators-to-actuators calibrations, meaning that if there are existing datasets on one sensor/actuator to calibrate the other sensor/actuator with small number of new data. For example, when calibrating soft sensors using few-shot learning, a possible approach is to define a kernel function that measures the similarity, between the source and target data, based on the characteristics of hysteresis. However, in soft robotic domain, it needs to be verified if these methods are applicable. Furthermore, existing studies in rigid robots that use Meta learning are primarily based on vision data; it may require a new problem definition that is suitable for soft sensors and actuators.

Second, although recent studies were focused on issues related to soft robots such as non-linearity and hysteresis, there are many other sources of error that have negative influences on their performances. Given that most of the soft sensors and actuators are fabricated via manual processes; in general, there are manufacturing errors within the same devices, which have an influence on the performances of machine learning algorithms. Although a sensor or actuator may be characterized using machine learning, it is unknown for the learned model to be applicable in different sensors or actuators due to the manufacturing errors. In addition, soft materials are generally slightly deformed after constant use; which has an influence on the performance of machine learning models. This results in a lack of generality, in that a machine learning method may require re-training upon the replacement of a device. One way to overcome this is to transfer the pre-trained parameters of the devices to the new devices or used devices to reduce re-training time [53, 54, 141, 142].

Third, the real-time applicability and limitations with respect to actual robots require further investigation. Several studies were recently conducted based on deep learning algorithms; however, calculations that incur significant computational loads are required, which can only be conducted using graphics processing units (GPUs). This increases the size of the computing device, thus increasing the robot size. Considering that many soft robots are mobile or wearable, an increase in size is not feasible. In addition, small-sized embedded devices do not allow for rapid calculations in real-time. Moreover, this increases the difficulty of robot control [101]. Recent studies conducted on artificial intelligence were focused on the optimization of machine learning models to increase the speed of calculations while maintaining the accuracy [81, 143], which can potentially overcome this limitation.

## 4. Conclusions

This review article presents existing machine learning approaches in the soft robotic research field. Machine learning algorithms are primarily applied to model the intrinsic non-linear characteristics of the soft materials. In several applications, the algorithms were used to obtain proprioception or current poses. In some other studies, machine learning was used to obtain human gestures/poses or for the control of soft actuators to realize the grasping of objects.

Machine learning algorithms have been used for the processing of soft sensor data for the realization of three main objectives, in addition to the solution of the abovementioned drawbacks of soft robots. First, learning algorithms such as kNN, ANN, CNN, and RNN were applied for the signal processing of individual soft sensors, soft tactile array sensors, and soft stretch sensors. Moreover, the algorithms could predict the location and magnitude of the pressure applied to the sensor, or estimate the shape of the structure in which the soft sensors were embedded. Finally, by integrating individual soft sensors, soft tactile array sensors and soft stretch sensors into robotic systems for sensing in practical uses, the collected sensor data were processed using machine learning algorithms such as SVM, DNN, and CNN, to obtain information on surroundings and related to the interaction between the robotics systems and objects.

Although soft actuators have the advantages of flexible motion with simple morphological structures, due to their versatility, there are limitations with respect to state modeling and the control of soft body systems. Control methods based on machine learning approaches such as FNN and RNN were developed for the following objectives: proprioception, model-based policy formulation for the control of soft actuators, and model-free policy formulation for the control of soft actuators. It should be noted that unlike other soft robotic applications, reinforcement learning algorithms have been extensively implemented to obtain policies for the control of robots.

Although there are several remaining limitations due to the large quantity of data required, additional unexpected error sources, and real-time measurements/controls; overall, machine learning algorithms are critical in soft robotics, as they can more effectively solve problems related to non-linearity and hysteresis when compared with traditional methods.
